# Effectiveness of behavior change in rehabilitation interventions to improve functional recovery after lower limb fracture: a systematic review

**DOI:** 10.1007/s12306-024-00845-x

**Published:** 2024-07-02

**Authors:** Christopher Bretherton, Ahmed Al-Saadawi, Fraser Thomson, Harbinder Sandhu, Janis Baird, Xavier Griffin

**Affiliations:** 1 Bone and Joint Health, Blizard Institute, Queen Mary University London, 4 Newark Street, London, E1 2AT UK; 2 Department of Trauma and Orthopaedic Surgery, Royal London Hospital, Barts Health NHS Trust, London, E1 1BB UK; 3 School of Medicine, Faculty of Medicine and Dentistry, Queen Mary University of London, London, England UK; 4 Oxford University Hospitals NHS Foundation Trust, Oxford, UK; 5 Division of Health Sciences, Warwick Clinical Trials Unit, University of Warwick, Coventry, CV4 7AL UK; 6 Centre for Developmental Origins of Health and Disease, University of Southampton, Southampton, SO17 1BJ UK

**Keywords:** Ankle fracture, Rehabilitation, Behaviour Change Technique

## Abstract

Ankle fractures are common injuries that can significantly impact mobility and quality of life. Rehabilitation following ankle fracture treatment is crucial for recovery, yet adherence to regimens remains a challenge. Behaviour Change Techniques (BCTs) have been suggested to improve adherence, but their effectiveness in ankle fracture rehabilitation is not well established. This review aimed to evaluate the effectiveness of BCTs in the rehabilitation of ankle fracture patients. We conducted a comprehensive search across multiple databases, including MEDLINE and EMBASE, focusing on Randomised Controlled Trials (RCTs) that incorporated BCTs into rehabilitation interventions. The effectiveness of BCTs on patient-reported outcomes (PROMs), quality of life, and adverse events was analysed. Nine RCTs met the inclusion criteria, encompassing a range of interventions that employed BCTs, most commonly including goal setting and instruction on how to perform behaviours, specifically physiotherapy exercises. The review found limited evidence supporting their effectiveness in improving PROMs. Only one study showed a significant positive effect, but it was deemed at high risk of bias. The lack of integration of behavioural theory in the design of rehabilitation interventions and the varied nature of the BCTs employed across studies may contribute to these findings. The use of BCTs in ankle fracture rehabilitation is prevalent, but this review highlights a significant gap their role of enhancing patient outcomes. Future research should incorporate a theory-based approach to intervention design, utilising a broader range of BCTs, to fully evaluate their potential in improving rehabilitation adherence and outcomes following ankle fracture .

**Supplementary Information:**

The online version contains supplementary material available at 10.1007/s12306-024-00845-x.

## Introduction

### Background

The ankle joint represents a common fracture site of the lower extremity, accounting for 14% of fracture-related hospital admissions in the United Kingdom [1]. Around 40% of ankle fractures are considered unstable, requiring surgical intervention to correct the deformity [2, 3]. Whilst most patients experience an 80–90% recovery of baseline ankle function following treatment, a sizeable proportion of patients continue to experience persistent pain and functional deficits leading to prolonged work absence and, in the long-term, to post-traumatic osteoarthritis and psychological consequences [4–6].

Rehabilitation is an essential component of ankle fracture care, enabling the patient to achieve a full recovery in function following surgical fixation [7]. Post-operative rehabilitation is one of the most heavily researched aspects of ankle fracture care, focusing chiefly on comparing device, manual or exercise therapies, and weight bearing strategies [8]. There is growing acknowledgement that promoting self-management with ankle exercises and adherence to physiotherapy regimens may improve outcomes. A recent meta-analysis conducted by Smeeing 2015 [9] illustrated that initiation of active ankle exercises and early weight bearing in the post-operative period was associated with an accelerated return to work and daily activities compared to patients with prolonged immobilisation strategies. The central issue is that many perceive that physiotherapy treatment only occurs during the physiotherapy clinic. The challenge remains to encourage patients to engage and continue physiotherapy regularly at home, for which there are various barriers such as anxiety, stress, and low self-efficacy [10].

Over the years, behaviour change theory has grown in popularity and a range of strategies have been developed to facilitate improved rehabilitation adherence in patients with ankle fractures [11, 12]. Michie et al. 2013 [13] developed the Behaviour Change Technique Taxonomy which consists of 93 distinct behavioural change techniques (BCT) clustered into 16 different groups. BCT is defined as an observable and reproducible component of a wider intervention that aims to facilitate behaviour change [13]. These behavioural interventions are proposed in the form of active ingredients. Some examples of BCTs include goal setting (i.e. running 1 mile everyday), problem solving (i.e. identifying barriers and creating strategies to overcome them), action planning (i.e. a plan of performing ankle exercises every day before going to work), and review of behaviour goals (i.e. assessing performance in relation to initial goals and whether any behavioural changes are needed) [13]. BCTs are beginning to show efficacy in treating musculoskeletal conditions with studies illustrating improved mobility and exercise adherence in patients with musculoskeletal disorders, but it has not been found to improve patient-reported outcomes. There has been limited investigation of behaviour change techniques in traumatic injury rehabilitation and none looking at ankle fracture recovery [14].

### Objectives

The primary aims of the systematic review are:

- To determine which behaviour change techniques (BCTs) have been most commonly used in studies comparing rehabilitation interventions after ankle fracture and determine which theories they are based on.
- To determine which BCTs are most effective in improving patient-reported outcomes after ankle fracture.

## Methods

The systematic review is reported according to the Preferred Reporting Items for Systematic Reviews and Meta-analyses (PRISMA) checklist [15]. A protocol for this systematic review was submitted to The International Prospective Register of Systematic Reviews (PROSPERO) on the 18th of March 2020 and was registered on the 9th June 2020 (PROSPERO: CRD42020170462) [16]. Medline, EMBASE, CINAHL, PsycINFO, AMED, CENTRAL, PEDRO and clinicaltrials.gov were searched from inception, using a search strategy developed with an information specialist. Searches were conducted on May 18th 2020 and repeated on March 2nd 2024. The Medline search strategy is included in the supplementary information and was modified for the other databases under the direction of the information specialist. Reference lists of included studies were searched. Unpublished and grey literature were not searched due to the reporting detail required to accurately code BCTs.

### Study selection

All titles and abstracts were imported to a reference manager database, and duplicates were removed. The remaining titles and abstracts were uploaded to Rayyan [17]. Two reviewers (CB and FT) independently screened the titles and abstracts against the inclusion criteria, scoring studies as "include", "exclude", or "maybe". All studies scored as "include" by either reviewer went forward for full-text review, and those scored as "maybe" were resolved by discussion. A third reviewer (XG) adjudicated any disagreement.

### Eligibility criteria

#### Inclusion

- Prospective randomised control trials (RCT), including pilot studies, that evaluated the effectiveness of BCTs on operatively and non-operatively ankle or hindfoot fractures in adult participants (aged 18 years or over) were eligible. Studies comparing different rehabilitation regimes against each other or against "usual care" were included. The control group could include BCTs, but the intervention group needed to incorporate additional BCTs.
- The interventions were any rehabilitation method using BCTs to improve patient-reported functional outcomes after ankle fracture. Only BCTs that provided sufficient detail of the components to allow them to be identified from the BCT taxonomy were included [13]. Interventions had to encourage active engagement for patients to continue the behaviour and not rely on the continued physical presence of a trainer or healthcare professional. The continued presence of peers or family support was permitted.

#### Exclusion

- Non-randomised or quasi-randomised studies, protocols or feasibility studies not reporting outcomes by treatment group, observational studies, cross-sectional studies, case series, case reports, abstracts, commentaries, and expert opinion studies.

### Outcome measures

The primary outcome was patient reported outcome measures (PROMs) of ankle or lower limb function. The secondary outcomes were quality of life and adverse events.

### Information sources

Full texts were sought through multiple sources, including OVID, PubMed, Search Oxford Libraries Online (SOLO), Library Hub Discover and WorldCat. Full-text articles were reviewed against the inclusion criteria. The intervention descriptions were reviewed according to and after completion of the BCT taxonomy training package [13]. Supplementary materials, protocols and intervention development papers were reviewed to look for underpinning behavioural theory and intervention descriptions for BCT coding. Authors were contacted via email to obtain missing data.

### Risk of *bias* assessment

The risk of bias was assessed using the Cochrane risk of bias tool [18]. This included assessment of: sequence generation, concealment of allocation, blinding, incomplete outcome data, selective reporting, and other sources [18].

### Analysis of studies

The review focused on describing the range of BCTs used and their effectiveness. Studies were summarised according to fracture location, management (operative vs non-operative treatment) and additional, non BCT interventions (e.g. immobilisation or weight-bearing restrictions). Studies were grouped based on the BCTs employed (according to the BCT taxonomy [13]) and where possible, their underlying behavioural theory.

## Summary measures and *meta*-analysis

Characteristics of studies were summarised as counts and percentages for categorical data and means and Standard Deviations (SDs) for continuous data. The Standardised Mean Difference (SMD) for included studies with available effect sizes and SDs were calculated and pooled using a random-effects model. Heterogeneity was investigated using I2, with an I2 of equal to or under 75% used as the cut-off for proceeding with meta-analysis [19]. Meta-analyses were undertaken using RevMan v.5.4 (Cochrane Collaboration, Vienna, Austria) and reported following PRISMA guidance.

A pre-planned sensitivity analysis included only studies at low risk of bias for the primary outcome. A further post-hoc sensitivity analysis was conducted to examine the risk of adverse events in operatively treated ankle fractures only.

### Certainty of evidence

The certainty of the evidence assessment was undertaken using the Grading of Recommendations, Assessment, Development and Evaluation (GRADE) approach and summarised using GRADEpro GDT Software [20, 21].

## Results

### Study selection

**Fig. 1:**
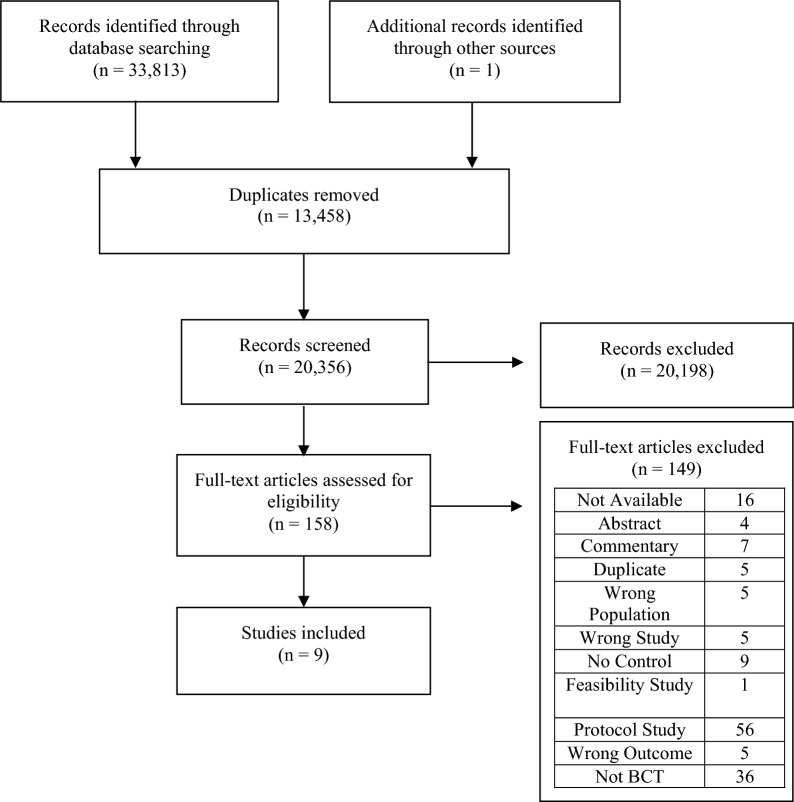
PRISMA flow diagram of study selection

The search results returned 33,814 items, with 20,356 remaining after removing duplicates. After the abstract screening, 158 full-text articles were sought. Sixteen full-text articles were unavailable; in all cases, the items were titles only and may have been excluded earlier in the searches if abstracts were available for review. Fifteen of the 16 unavailable items were published in non-English-language journals, and 14 of the 16 were published pre-1990. After exclusion criteria, nine studies remained and were included in the final review. Figure 1 shows a PRISMA flow diagram detailing the selection process, including reasons for exclusion at full-text review.

### Included studies and characteristics

#### Population and setting

Nine studies were included, which enrolled a total of 1,437 patients. Two took place in Australia [22, 23], two in Canada [24, 25], two in Sweden [26, 27], two in the UK [28, 29], and one in the Republic of Ireland [30]. Six studies focused only on operatively treated ankle fractures [24–27, 29, 30] and three on non-operatively and operatively treated ankle fractures [22, 23, 28]. No studies of hindfoot fractures met the inclusion criteria.

#### Behavioural theory and interventions

No studies referenced behavioural theory in their intervention design or description. Instead, BCTs were coded from the published articles and protocols as described in the methods section. The target behaviour in eight of the studies was ankle exercises [23–30]. In addition to the BCTs used, five studies had additional, non-BCT interventions. In four studies, the intervention group received some form of removable splint, and the control group were immobilised in a plaster cast [27–30]. In one study, the intervention group was permitted early weight-bearing in a removable splint and the control group had their weight-bearing restricted in a plaster cast [26].

Eight studies compared one intervention group to one control group [23–30]. One study, Moseley 2005 [22], consisted of three groups: an exercise-only group, a short-duration stretch group (six minutes per day) and a long-duration stretch group (30 min per day). For the meta-analyses, just the long stretch group was compared to the control group due to data availability. A summary of study characteristics is provided in Table 2.

#### Behaviour change techniques

A total of 34 BCTs were coded throughout the nine included studies, which included eight unique behaviour change techniques. In six studies [24, 25, 27–30], "4.1 instruction on how to perform a behaviour" was coded as the intervention referring to a verbal or written instruction to perform ankle exercises. Four studies [24, 28–30] combined this instruction with a specific, numeric daily exercise repetition goal and thus, "1.1 goal setting (behaviour)" was coded in addition. Furthermore, two studies [24, 30] reported that these instructions were delivered by a dedicated physiotherapist and so “9.1 credible source” was coded. Mayich 2013 [25] also provided a leaflet with educational advice coded as “5.1 information about health consequences”. In Moseley 2015 [23], the control group received an exercise and advice leaflet provided by a physiotherapist in fracture clinic; accordingly, the BCTs coded were "1.1 goal setting (behaviour)", "4.1 instruction on how to perform a behaviour", and "9.1 credible source". The intervention group in addition undertook a supervised physiotherapy programme. The additional BCTs coded in the intervention group were "8.1 behavioural practise/ rehearsal", "8.6 generalisation of target behaviour" and "8.7 graded tasks". The coded BCTs in Moseley 2005 [22] include "1.1 goal setting (behaviour)", "4.1 instruction on how to perform a behaviour", "8.1 behavioural practise/ rehearsal", "8.6 generalisation of target behaviour", "8.7 graded tasks" and "9.1 credible source". In Nilsson 2009 [26], a physiotherapist-led training programme centred around ankle exercises led to BCT coding of "1.1 goal setting (behaviour)", "4.1 instruction on how to perform a behaviour", "8.1 behavioural practise/ rehearsal", "8.6 generalisation of target behaviour", "8.7 graded tasks" and "9.1 credible source". Additionally, "1.2 goal setting (outcome)" and “5.1 information about health consequences” were also coded for this study. Table 1 shows the most commonly coded BCTs, with percentages corresponding to the proportion of all studies using the stated BCT.

**Table 1:** Summary of coded behaviour change techniques in included studies

Behaviour Change Technique (BCT)	N (%^1^)
4.1 Instruction on how to perform behaviour [22–30]	9 (100%)
1.1 Goal setting (behaviour) [22–26, 28, 29]	8 (89%)
9.1 Credible source [22–24, 26, 30]	5 (56%)
8.1 Behavioural practice / rehearsal [22, 23, 26]	3 (33%)
8.6 Generalisation of target behaviour [22, 23, 26]	3 (33%)
8.7 Graded tasks [22, 23, 26]	3 (33%)
5.1 Information about health consequences [25, 26]	2 (22%)
1.2 Goal setting (outcome) [26]	1 (11%)
^1^% of all included studies using this BCT: they are not mutually exclusive	

### Risk of *bias*

**Fig. 2:**
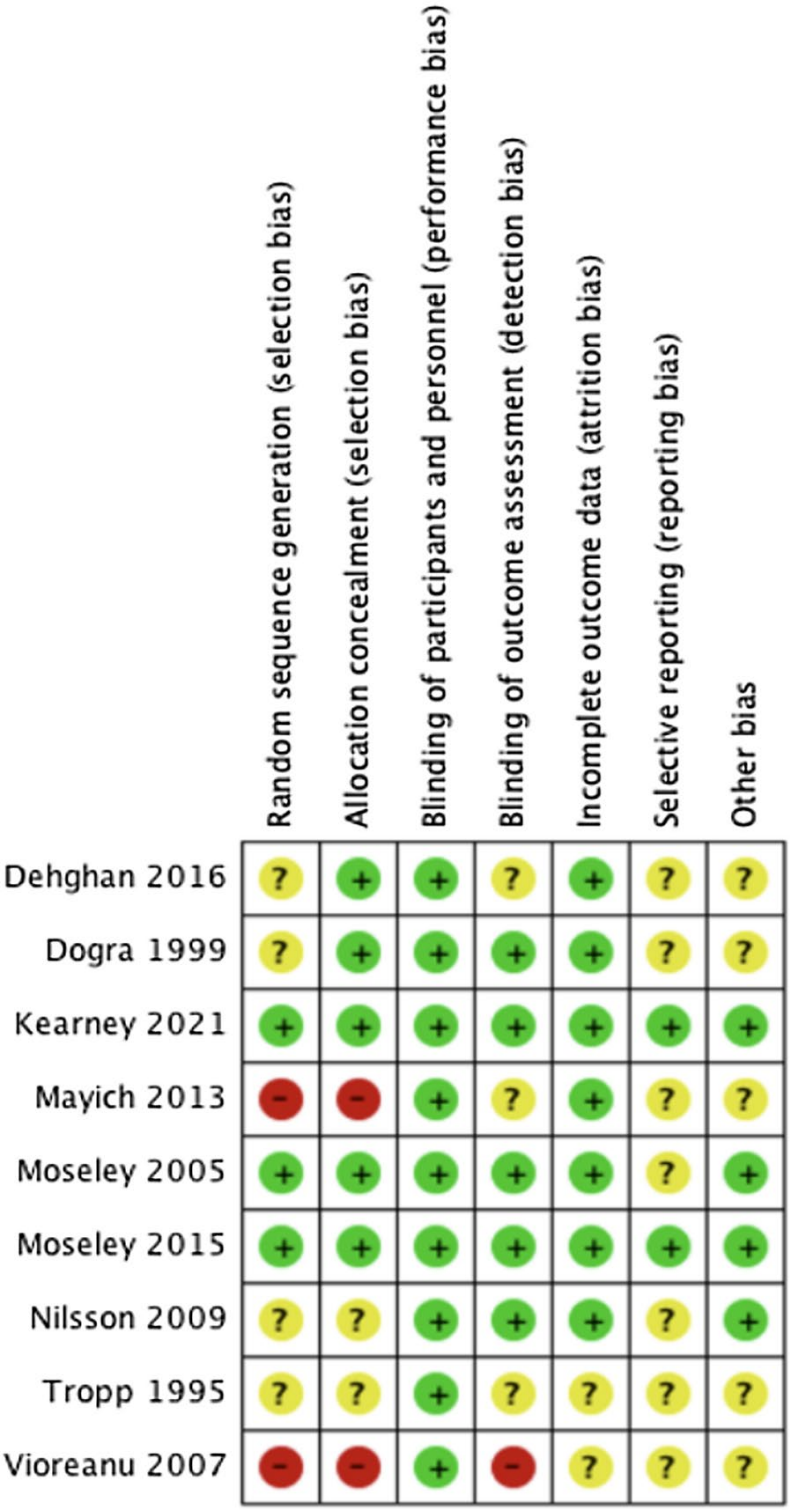
Risk of bias summary

A summary risk of bias table for all nine included studies is provided in Fig. 2. Kearney 2021 [28], Moseley 2005 [22] and Moseley 2015 [23] were judged as a low risk of selection and allocation bias. Nilsson 2009 [26] and Tropp 1995 [27] provided insufficient information and were therefore judged as an unclear risk of bias. Dehghan 2016 [24] and Dogra 1999 [29] were also judged as a low risk of selection bias but did not describe the sequence generation process and therefore were deemed as unclear risk. Mayich 2013 [25] and Vioreanu 2007 [30] incurred a high risk of selection and allocation bias as it used the odd / even hospital number and date of birth method respectively for allocation. All studies were judged as low risk of bias for blinding of participants and personnel because it was determined that the interventions (exercises and stretching) were unable to be blinded. Dogra 1996 [29], Kearney 2021 [28], Moseley 2005 [22], Moseley 2015 [23] and Nilsson 2009 [26] were deemed at low risk of detection bias. On the contrary, Dehghan 2016 [24], Dogra 1999 [29] and Tropp 1995 [27] did not state who assessed outcomes and were deemed as unclear risk, and Vioreanua 2007 [30] stated that "one of the authors" assessed clinical outcomes, so this was judged to be at high risk of bias. Furthermore, Dehghan 2016 [24], Dogra 1999 [29], Mayich 2013 [25], Moseley 2005 [22] and Nilsson 2009 [26] had similar rates of dropout between groups and > 85% follow-up rates and so were judged as low risk of bias for outcome data. Tropp 1995 [27] and Vioreanua 2007 [30] did not clearly report the completeness of outcome data and so were judged as unclear risk of bias. Moseley 2015 [23] and Kearney 2021 [28] were deemed as low risk of bias for selective reporting, whilst the remaining studies had an unclear risk. Kearney 2021 [28], Moseley 2005 [22] and Moseley 2015 [23] were judged as low risk as no other important sources of bias were identified. While Nilsson 2009 [26] had uncertainty around the randomisation process, there was adequate detail in the methods and reporting to judge the study as low risk of other sources of bias. The remaining studies were judged as unclear risk as there was limited reporting of baseline characteristics and outcomes to enable assessment.

### Synthesis of results

#### Patient-reported outcomes

Six studies used a PROM of lower limb function as the primary outcome. The primary outcome used by Moseley 2005 [22] and Moseley 2015 [23] was the Lower Extremity Functional Scale (LEFS) at three months post-injury. The primary outcome for Kearney 2021 [28], Nilsson 2009 [26], Tropp 1995 [27] and Vioreanu 2007 [30] was the Olerud and Molander Ankle Score (OMAS) at 16 weeks, six months, 12 months and 12 weeks, respectively.

The primary outcome for Dehghan 2016 [24] was return to work, for Mayich 2013 [25] it was satisfaction with staff and for Dogra 1999 [29] the primary outcome was not stated. Dehghan 2016 [24] reported OMAS at six weeks and three, six and 12 months postoperatively. Dogra 1999 [29] and Mayich 2013 [25] reported OMAS at 12 weeks and three months post-operatively, respectively. Only Vioreanu 2007 [30] found a statistically significant difference in the patient-reported outcome measurements, favouring the BCT intervention group.

#### *Meta*-analysis of patient-reported functional outcomes

Four studies reported sufficient results to include and calculate SMDs for patient-reported functional outcomes. After contacting all authors, data for two further studies was obtained, bringing the total number of studies included in the meta-analysis to six. Heterogeneity was considerable, but within threshold when including all six studies (I2 = 75%). When removing Vioreauna 2007 [30], which was judged as high risk of bias, heterogeneity was reduced substantially (I2 = 0%). Of note, the mean OMAS in both treatment groups in Vioreanu 2007 [30] was considerably higher and the SD substantially lower than in other published studies in this population [28, 31]. This can be seen in Table 2. This may indicate that the patient populations or conduct of the study were atypical.

**Table 2:** Summary of included studies

Study / setting	Population	Target behaviour and time frame	Behaviour change techniques	Additional non-BCT interventions	PROM / effect size
Dehghan 2016, Canada, RCT	Operatively treated ankle fractures Participants: ‡I: 56, †C: 54 Mean age: ‡I: 42.1 years, †C: 41.5 years	Ankle exercises 2–6 weeks post-operatively. Physiotherapist delivered	‡I: 1.1 goal setting (behaviour), 4.1 instruction on how to perform a behaviour, 9.1 credible source †C: nil	‡I: Splint, EWB †C: Plaster, DWB	OMAS at 3 months ‡I: 62 (SD 22), †C: 56 (SD 21). Effective: No Mean difference not reported
Dogra 1999, UK, RCT	Operatively treated ankle fractures. Participants: ‡I: 26, †C: 26 Mean age: 42.7 years	Ankle range of motion exercises From 24 h postoperatively	‡I: 1.1 goal setting (behaviour), 4.1 instruction on how to perform a behaviour †C: nil	‡I: Removable plaster †C: Plaster	OMAS at 3 months ‡I: 46.2 (SD not reported), †C: 43.4 (SD not reported) Effective: No Mean difference not reported
Kearney 2021, UK, RCT	Operatively and Non-operatively treated ankle fractures. Participants: ‡I: 335, †C: 334 Mean age: 46 years	Ankle exercises. First 6 weeks post-injury or surgery, for a minimum of 3 weeks. Educational handout	‡I: 1.1 goal setting (behaviour), 4.1 instruction on how to perform a behaviour †C: nil	‡I: Removable brace †C: Plaster	OMAS at 16 weeks ‡I: 64.5 (SD 22.4), †C: 62.4 (SD 23.4). Effective: No Mean difference 2.1 (CI -1.9 to 6.2)
Mayich 2013, Canada, RCT	Operatively treated ankle fractures. Participants: ‡I: 20, †C: 20 Mean age: ‡I: 39.9 years, †C: 34.8 years	Educational handout and physiotherapy exercises. 2–6 weeks postoperatively. Physiotherapist delivered	‡I: 1.1 goal setting (behaviour), 4.1 instruction on how to perform a behaviour, 5.1 information about health consequences †C: nil	N/A	OMAS at 3 months ‡I: Not reported, †C: Not reported. Effective: No Mean difference not reported
Moseley 2005, Australia, RCT	Operatively and Non-operatively treated ankle fractures. Participants: ‡I short: 49, ‡I long: 51, C: 50 Mean age: ‡I short: 43 years, ‡I long: 47 years, †C: 49 years	Supervised manual stretching program. After removal of plaster cast. Physiotherapist delivered	‡I: 1.1 goal setting (behaviour), 4.1 instruction on how to perform a behaviour, 8.1 behavioural practise/rehearsal, 8.6 generalisation of target behaviour, 8.7 graded tasks, 9.1 credible source †C: The same BCTs used for ankle exercises, but nil for stretching	N/A	LEFS at 3 months ‡I short: 67.4 (SD 11.1), ‡I long: 65.8 (SD 12.9), †C: 65.8 (SD 13.6) ‡I long vs C: Effective: No. Mean difference 0.9 (CI -4.7 to 6.6)
Moseley 2015, Australia, RCT	Operatively and Non-operatively treated ankle fractures. Participants: ‡I: 106, †C: 108 Mean age: ‡I: 43.0 years, †C: 41.3 years	Supervised exercise program. After removal of cast	‡I: 1.1 goal setting (behaviour), 4.1 instruction on how to perform a behaviour, 8.1 behavioural practise/rehearsal, 8.6 generalisation of target behaviour, 8.7 graded tasks, 9.1 credible source †C: 1.1 goal setting (behaviour), 4.1 instruction on how to perform a behaviour, 9.1 credible source	N/A	LEFS at 3 months ‡I: 64.3 (SD 15.1), †C: 64.3 (SD 13.5). Effective: No Mean difference 0.4 (CI -3.3 to 4.1)
Nilsson 2009, Sweden, RCT	Operatively treated ankle fractures. Participants: ‡I: 52, †C: 58 Mean age: Male: 37 years, Female: 48 years	Ankle exercises. After removal of cast (mean 43 days post-operatively). Physiotherapist delivered	‡I: 1.1 goal setting (behaviour), 1.2 goal setting (outcome), 4.1 instruction on how to perform a behavior, 5.1 information about health consequences, 8.1 behavioral practise/rehearsal, 8.6 generalisation of target behaviour, 8.7 graded tasks, 9.1 credible source †C: nil	N/A	OMAS at 6 months ‡I: 62.4 (SD 25.1), †C: 63.5 (SD 20.9). Effective: No Mean difference not reported
Tropp 1995, Sweden, RCT	Operatively treated ankle fractures. Participants: ‡I: 15, †C: 15 Mean age: 26 years	Ankle exercises. First 6 weeks postoperatively. Physiotherapist delivered	‡I: 4.1 instruction on how to perform a behavior †C: nil	‡I: Brace †C: Plaster	OMAS at 10 weeks ‡I: Not reported, †C: Not reported. Effective: No Mean difference not reported
Vioreanu 2007, Republic of Ireland, RCT	Operatively treated ankle fractures. Participants: ‡I: 33, †C: 29 Mean age: ‡I: 37.2 years, †C: 34.9 years	Ankle exercises. First 6 weeks Postoperatively. Physiotherapist delivered	‡I: 1.1 goal setting (behavior), 4.1 instruction on how to perform a behavior, 9.1 credible source †C: nil	‡I: Removable plaster †C: Plaster	OMAS at 12 weeks ‡I: 93.2 (SD 8.8), †C: 81.1 (SD 9.6). Effective: Yes Mean difference not reported

^‡^I: Intervention Group, †C: control group, Mean diff: mean difference (effect size), RCT: Randomised Controlled Trial, OMAS: Olerud and Molander Ankle Score, LEFS: Lower Extremity Functional Score

**Fig. 3:**
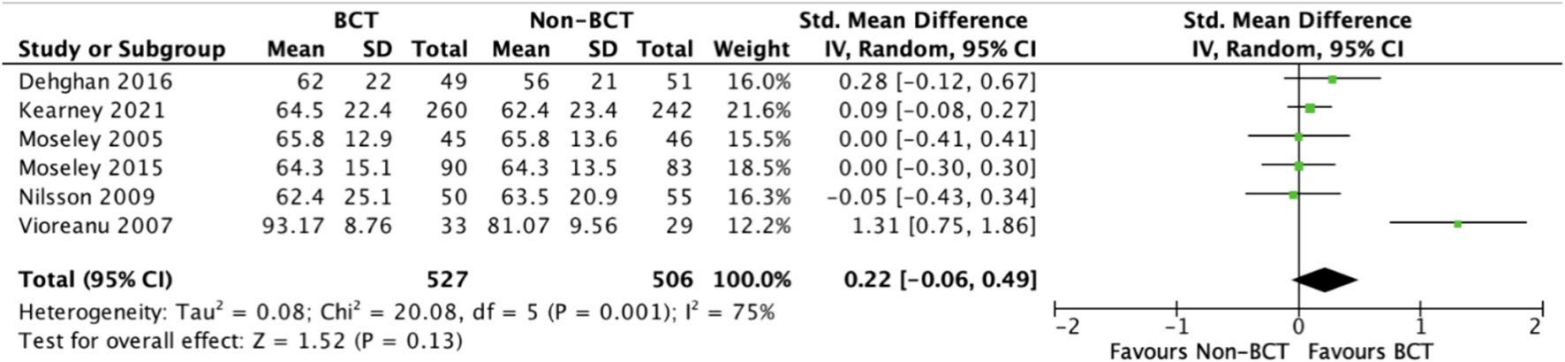
A forest plot comparing patient-reported functional outcomes

The pooled SMD for Dehghan 2016 [24], Kearney 2021 [28], Moseley 2005 [22], Moseley 2015 [23], Nilsson 2009, [26] and Vioreanu 2007 [30] was 0.22 (CI -0.06 to 0.49), favouring the BCT group (non-significant). A forest plot is included in Fig. 3. There remained an insignificant difference between the BCT (intervention) and non-BCT (control) groups when Vioreanu 2007 [30] was excluded: 0.07 (CI -0.06 to 0.20) in favour of the BCT group (non-significant).

#### *Meta*-analysis of quality of life

**Fig. 4:**
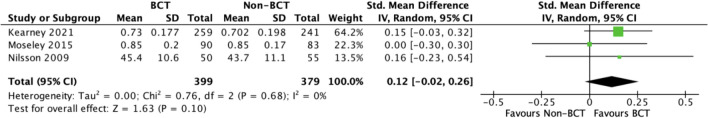
A forest plot comparing quality of life

Two studies reported a global quality of life score [23, 28]. Kearney 2021 [28] reported the EQ-5D-5L [32] at 16 weeks and Moseley 2015 [23] reported the Assessment of Quality of Life Instrument [33] at three months. Three studies [24, 26, 30] reported the Short-Form 36 (SF-36); this is split into a Mental and Physical component, and combining them into a global score for meta-analysis is not advised [34]. No significant differences were found in the studies that reported SF-36, and only Nilsson 2009 [26] reported the mean scores with SDs in each group (at 6 months) to enable inclusion in the meta-analysis. For the purposes of the meta-analysis, the physical component of the SF-36 was included, as physical quality of life was prioritised for this review. The remaining studies did not report a quality-of-life score [22, 25, 27, 29]. The pooled standardised mean difference for Kearney 2021 [28], Moseley 2015 [23] and Nilsson 2009 [26] was 0.12 (CI -0.02 to 0.26) in favour of the BCT group (non-significant). Figure 4 shows the forest plot for quality of life.

#### *Meta*-analysis of adverse events—all

**Fig. 5:**
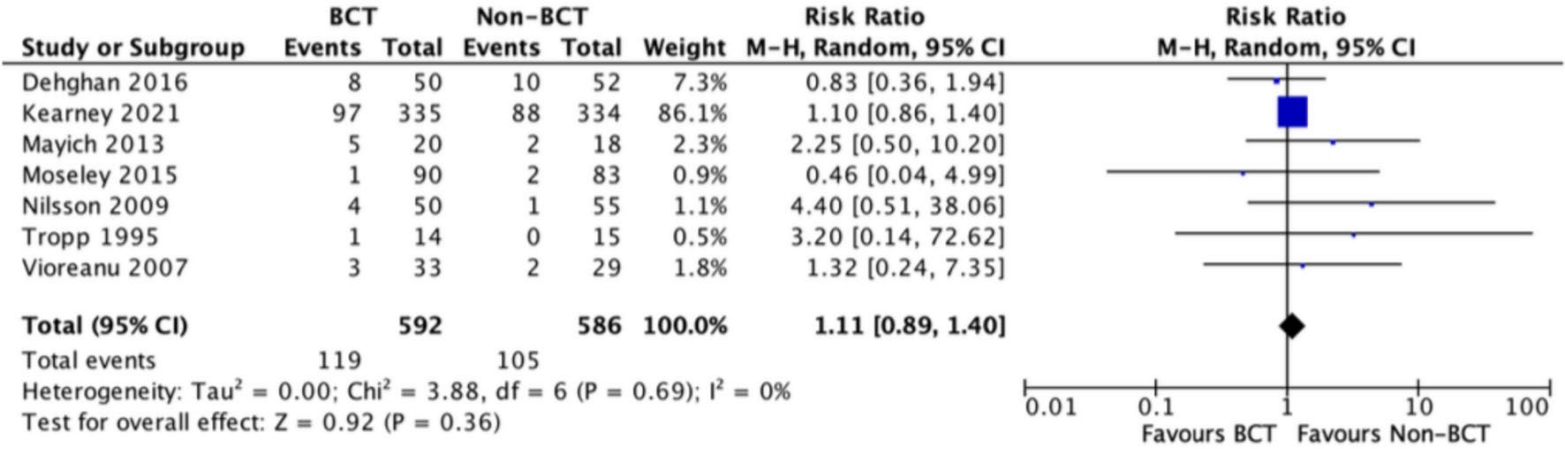
A forest plot showing the risk of adverse events

Complications were noted in eight studies, with Moseley 2005 [22] finding no complications in study participants. Dogra 1999 [29] reported a superficial wound infection, but it was not specified which treatment group this occurred in, so it was excluded from the meta-analysis. Commonly reported complications throughout the studies included Deep Vein Thrombosis (DVT) or Pulmonary Embolus (PE), infection or wound healing complications and re-operation. The results of all complications are displayed in Fig. 5, reporting a non-statistically significant risk ratio (RR) favouring the control group of 1.11 (CI 0.89 to 1.40).

#### Post-hoc analysis: *meta*-analysis of adverse events—operatively treated

**Fig. 6:**
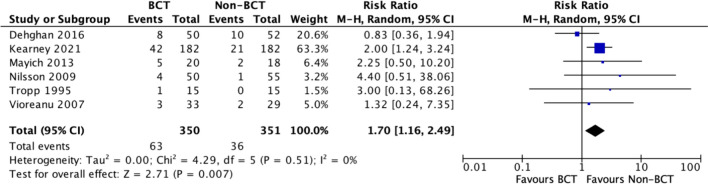
A forest plot showing the risk of adverse events for operatively treated ankle fractures only

On inspection of the complications in all studies (excluding numbness), 94/136 (69.1%) were complications unique to surgery, including infection, wound healing problems and re-operation. Kearney 2021 [28] reported complications unique to the surgical group, and so a revised meta-analysis including only surgical complications from this study is provided in Fig. 6 (which also excludes Moseley 2015 [23], which reported operatively and non-operatively treated patients together). Other complications that can occur in operatively treated and non-operatively treated patients were not included in the reporting. This is important to consider as these could have a higher incidence in the control group. However, these were similar in the BCT and non-BCT groups in Kearney 2021 [28], with identical rates of DVT, PE and complex regional pain syndrome (CRPS), with one extra non-union in the BCT group and four extra pressure sores in the non-BCT group. In the revised meta-analysis, the RR for a complication occurring was 1.70 (CI 1.16 to 2.50, p = 0.007) for patients in the BCT group.

#### Certainty of evidence

**Fig. 7:**
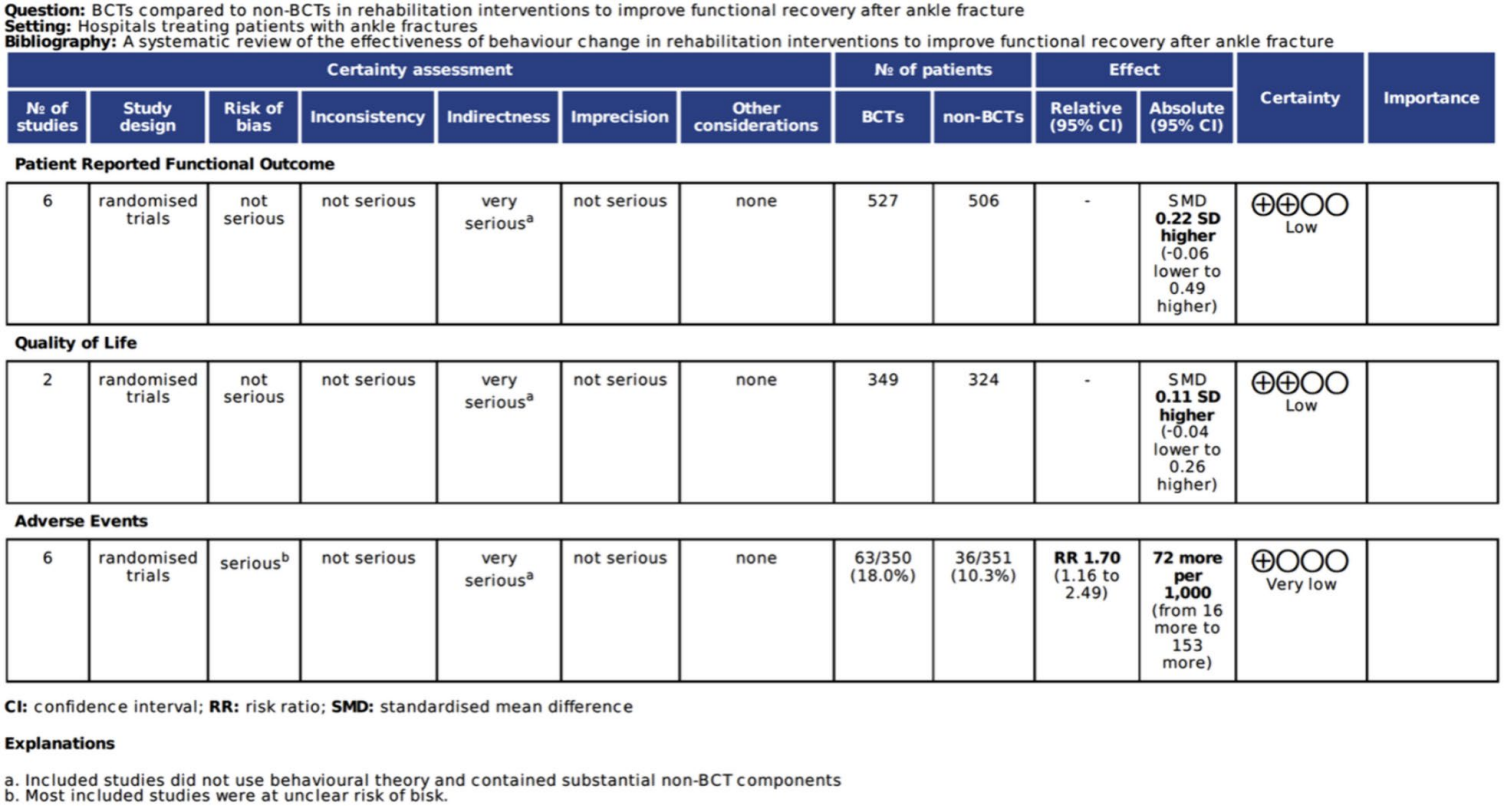
GRADE summary of findings

Figure 7 provides an assessment of the certainty of the evidence for the reported outcomes using the GRADE approach [21].

## Discussion

There was low-certainty evidence from six studies [22, 23, 26, 28] that the use of BCTs did not significantly impact patient-reported functional outcomes after ankle fracture. Two of these studies [23, 28] also found that the use of BCTs did not significantly impact participant’s quality of life after ankle fracture. Nilsson 2009 [26] reported that subjects under 40 years in the intervention group scored a higher OMAS score than those above 40 years (P = 0.028). The typical physiotherapy interventions applied in ankle fracture rehabilitation are generally not adapted towards the needs of older adults [35]. It is not uncommon for elderly patients to suffer from mobility and balance problems and tailoring physiotherapy interventions towards these limitations, as opposed to routine ankle rehabilitation exercises, can enhance their functional capacity [35–37]. This represents a wider issue across the current studies assessing physiotherapy as a rehabilitative intervention, and formulating an individualised regimen for each patient based on their needs may lead to improved recovery outcomes. Moreover, a statistically significant proportion of patients in the control groups of Nilsson 2009 [26] and Moseley 2015 [23] engaged in physiotherapy regimens, independently of the trial, possibly interfering with the aforementioned outcomes. However, post-hoc sensitivity analysis by Moseley 2015 [23] revealed that control group engagement in physiotherapy had no significant effect on the overall effect.

The review demonstrated a lack of behavioural theory used in the design of rehabilitation interventions after ankle fracture. Instead, the existing use of BCTs in this area centred around conventional methods of physiotherapy delivery: instruction and encouragement to perform ankle exercises. When developing a complex intervention, using a theory-based approach can enhance outcomes as it allows for the identification of the causal factors driving a particular behaviour, and this insight can subsequently be utilised to develop interventions that target the aforementioned [38]. Studies have highlighted variables such as low mood, low self-efficacy, and inadequate social support are associated with poor outcomes following surgery including chronic pain, long-term unemployment, and ultimately a worse quality of life [10, 39, 40]. Developing interventions that incorporate BCTs focusing on these areas, for example, may enhance their overall functional recovery, in comparison to exclusively implementing physiotherapy interventions where, as this review has found, effectiveness is unclear. Nonetheless, these conventional BCTs are also being used in current musculoskeletal interventional studies [35, 41], reinforcing their likely acceptability and indicating they could form the baseline level of therapy in the design of future interventions in this area. Ankle fracture rehabilitation could seek to build on these and include a broader range of BCTs, which have proven efficacious in non-acute musculoskeletal conditions [42, 43].

Six studies [24–28, 30] demonstrated that the use of BCTs significantly increased the risk of an adverse event after operatively-treated ankle fracture (RR 1.7 (CI 1.16 to 2.49)). This equates to 72 more complications per 1,000 patients treated (16 to 153). However, this is unlikely to represent an issue with BCTs because the studies had substantial non-BCT components, and five studies [24–27, 30] were at unclear or high risk of bias, thereby hindering the certainty of the observed outcomes. A systematic review by Sernandez 2021 [44] illustrated that early initiation of weight bearing and rehabilitation following operative ankle fractures is safe as it was not associated with a significant increase in complications in comparison to a delayed weight bearing approach. However, *immediate* mobilisation of the ankle may interfere with wound healing, and subsequently increase the risk of wound-related complications [44]. Nonetheless, it does highlight a broader consideration for the rehabilitation of patients after ankle fracture surgery. There is a trend toward promoting earlier movement and weight-bearing after ankle fracture surgery, with evidence suggesting this leads to an earlier return to work and hobbies [7, 24]. These early functional improvements may obscure an increased rate of complications, particularly wound healing problems and infection. Most studies have been powered to detect a change in PROMs, not complications [24, 35]. Thus, they may be insufficiently powered to detect significant differences in adverse events. One study comparing different immobilisation and weight-bearing strategies even stopped recruitment early due to superior PROMs in the early movement and weight-bearing group [45]. The largest single study in this area inadvertently masked this issue by combining operative and non-operatively treated populations [24]. Future studies using BCTs to promote early movement should look carefully at the risk of wound healing complications and consider including additional BCTs to enable monitoring, detection and avoidance of these complications.

The systematic review has used a comprehensive taxonomy to identify and code BCTs [13]. This will enable faithful replication of the behavioural components of the interventions for other researchers in the future. The broad search strategy also provides confidence that all relevant studies, including a wide spectrum of possible rehabilitation interventions, have been included.

The central limitation of this review is that it has been unable to answer the research questions due to the lack of behavioural theory used in the design of the included study interventions, as discussed previously. This limitation is the primary reason there is low certainty evidence for the outcomes examined: there were substantial non-BCT components testing different physiotherapy, immobilisation and weight-bearing strategies rather than discrete, theory-based BCTs. The interventions were designed to simply provide instructions rather than influence the psychological motivations of participants to engage with the behaviours.

## Conclusion

There were a range of BCTs identified in the rehabilitation interventions of patients with ankle fractures. The most commonly coded BCTs were: 4.1 instructions on how to perform a behaviour and 1.1 goal setting (behaviour), both of which are centred around the delivery of physiotherapy exercises. The use of BCTs did not significantly improve patient-reported outcomes in all studies, but one, which was found to have a high risk of bias. The lack of behavioural theory used in the design of rehabilitation interventions after ankle fracture has limited the usefulness of this review to meet the objectives. It highlights the need for more studies to incorporate behavioural theory into interventions to improve their effectiveness, and ultimately assess the true potential of BCTs in the rehabilitation stage of ankle fractures.

## Supplementary Information

Below is the link to the electronic supplementary material.

Supplementary file1 (DOCX 21 KB)
