# Supplementary material for: Effectiveness of behavior change in rehabilitation interventions to improve functional recovery after lower limb fracture: a systematic review
Source: Musculoskelet Surg. 2024 Jul 2;108(4):417–29. doi: 10.1007/s12306-024-00845-x (PMC11582149; doi:10.1007/s12306-024-00845-x)
Supplement: Supplementary file 1 — Supplementary file1 (DOCX 21 KB) [file 12306_2024_845_MOESM1_ESM.docx]

| **MEDLINE Search Strategy**  1. exp Lower Extremity/ |
| --- |
| 2. lower extremit*.ti,ab. |
| 3. lower limb*.ti,ab. |
| 4. foot.ti,ab. |
| 5. feet.ti,ab. |
| 6. (hip or hips).ti,ab. |
| 7. (knee or knees).ti,ab. |
| 8. (leg or legs).ti,ab. |
| 9. (thigh or thighs).ti,ab. |
| 10. (ankle or ankles).ti,ab. |
| 11. forefoot.ti,ab. |
| 12. (heel or heels).ti,ab. |
| 13. exp "Bones of Lower Extremity"/ |
| 14. femur*.ti,ab. |
| 15. femora*.ti,ab. |
| 16. tibia*.ti,ab. |
| 17. metatarsal*.ti,ab. |
| 18. tarsal*.ti,ab. |
| 19. ((toe or toes) adj3 phalan*).ti,ab. |
| 20. patella*.ti,ab. |
| 21. pelvi*.ti,ab. |
| 22. acetabul*.ti,ab. |
| 23. 1 or 2 or 3 or 4 or 5 or 6 or 7 or 8 or 9 or 10 or 11 or 12 or 13 or 14 or 15 or 16 or 17 or 18 or 19 or 20 or 21 or 22 |
| 24. exp Fractures, Bone/ |
| 25. fractur*.ti,ab. |
| 26. (broke or broken or break*).ti,ab. |
| 27. 24 or 25 or 26 |
| 28. 23 and 27 |
| 29. exp Rehabilitation/ |
| 30. rehab*.ti,ab. |
| 31. exp Physical Therapy Modalities/ |
| 32. physiotherap*.ti,ab. |
| 33. physical therap*.ti,ab. |
| 34. exercis*.ti,ab. |
| 35. physical activit*.ti,ab. |
| 36. 29 or 30 or 31 or 32 or 33 or 34 or 35 |
| 37. 28 and 36 |
